# Juvenile Idiopathic Arthritis-Associated Uveitis: A Nationwide Population-Based Study in Taiwan

**DOI:** 10.1371/journal.pone.0070625

**Published:** 2013-08-05

**Authors:** Hsin-Hui Yu, Pau-Chung Chen, Li-Chieh Wang, Jyh-Hong Lee, Yu-Tsan Lin, Yao-Hsu Yang, Chang-Ping Lin, Bor-Luen Chiang

**Affiliations:** 1 Department of Pediatrics, National Taiwan University Hospital, Taipei, Taiwan; 2 Institute of Occupational Medicine and Industrial Hygiene, National Taiwan University College of Public Health, Taipei, Taiwan; 3 Department of ophthalmology, National Taiwan University Hospital, Taipei, Taiwan; 4 Department of Medical Research, National Taiwan University Hospital, Taipei, Taiwan; University of Leuven, Rega Institute, Belgium

## Abstract

**Objective:**

The incidence and prevalence of juvenile idiopathic arthritis (JIA) vary widely across the world but data in East Asia is lacking. Uveitis is a serious cause of morbidity in JIA. This study aimed to analyze the incidence and prevalence of JIA, and the characteristics of JIA-associated uveitis in Taiwan.

**Methods:**

A population-based cohort study was conducted using the Taiwan National Health Insurance Research Database. Each patient was individually tracked from 1999 to 2009 to identify the diagnosis of JIA and uveitis using the International Classification of Diseases diagnostic codes. Multivariate logistic regression was used to determine the risk factors and complications of uveitis in patients with JIA.

**Results:**

The study cohort had 2636 cases of JIA and included juvenile rheumatoid arthritis (57.7%), enthesitis-related arthritis (ERA) (39.2%), and psoriatic arthritis (3.1%). The average annual incidence of JIA and JIA-associated uveitis were 4.93 (range, 3.93–6.23) and 0.25 (range, 0.12–0.37) cases per 100,000 population, respectively. The average period prevalence of JIA was 33.8 cases per 100,000 population. Uveitis occurred in 4.7% of patients with JIA, while JIA-associated uveitis was complicated by cataract (11.2%) and glaucoma (24.8%). Enthesitis-related arthritis was significantly associated with uveitis (OR: 3.47; 95% CI: 2.24–5.37) (*p*<0.0001). Uveitis diagnosed before JIA was the most significant risk factor for complications of glaucoma or cataract (OR: 3.54; 95% CI: 1.44–8.72) (*p* = 0.006).

**Conclusions:**

The incidence of JIA is low but that of JIA-associated uveitis is increasing. Higher percentage of males in patients with ERA and the strong association between ERA and uveitis are unique for children with JIA in Taiwan. Uveitis diagnosed before arthritis is an important risk factor for complications. Continuous ophthalmologic follow-up is needed for children with JIA or uveitis of unknown etiology.

## Introduction

Juvenile idiopathic arthritis (JIA) is the most common chronic rheumatic disease among children. It collectively refers to a group of chronic arthritis of at least six weeks duration in children or adolescents under the age of 16. The 1997 International League of Associations for Rheumatology (ILAR) criteria defined seven subtypes of JIA based on clinical and laboratory parameters: systemic JIA, oligoarticular JIA, rheumatoid factor (RF)-negative polyarticular JIA, RF-positive polyarticular JIA, enthesitis-related arthritis (ERA), psoriatic arthritis (PsA), and undifferentiated JIA [1]. The ILAR criteria for JIA replaced the 1977 American College of Rheumatology (ACR) criteria for “juvenile rheumatoid arthritis (JRA)” [2] and the 1977 European League Against Rheumatism (EULAR) criteria for “juvenile chronic arthritis (JCA)” [3]. The reported incidence and prevalence of JIA vary widely across the world, partly due to its heterogeneous nature and multi-factorial pathogenesis, which is influenced by genetic and environmental factors [4].

The most common extra-articular manifestation of JIA is uveitis. The intra-ocular inflammation primary affects the iris and ciliary body (iridocyclitis or anterior uveitis). Uveitis is reported in 5.6–24.4% of patients with JIA, especially those with the oligoarthritis type (10–30%) [5], [6]. In oligoarticular JIA, uveitis often has a chronic, asymptomatic, and insidious onset. Risk factors include anti-nuclear antibody (ANA) positivity, young age at onset of arthritis (≤6 years), female sex, and oligoarthritis [7]. On the other hand, acute symptomatic anterior uveitis in ERA, which has its counterpart in HLA-B27-associated acute anterior uveitis in adults, carries a better prognosis [5]. Patients with systemic JIA, RF-positive polyarthritis, and PsA rarely develop uveitis. Morbidity of JIA-associated uveitis includes cataracts, glaucoma, band keratopathy, and loss of vision. Outcome has improved over the past 20 years, but for children with JIA, which accounts for 5–81.5% of pediatric uveitis cases, it remains a serious cause of morbidity and loss of vision [8].

To date, there has been no large-scale epidemiologic study involving long-term follow-up of JIA and JIA-associated uveitis in Asia. The aim of this population-based study was to estimate the annual incidence, prevalence, and principal characteristics of children with JIA in Taiwan using the National Health Insurance research database (NHIRD). Complications of JIA-associated uveitis were also investigated in a long-term follow-up of children with JIA.

## Materials and Methods

### Ethics Statement

This National Health Research Institute (NHRI) Ethics Review Committee approved the study. The review board waived the requirement for written informed consent from the patients, parents, caretakers, or guardians because the secondary data were de-identified and stripped of personal information prior to use.

### Database

The sampling cohort dataset was obtained from the Taiwan National Health Insurance Research Database (NHIRD) based on the National Health Insurance Program (NHIP) that was implemented in March 1995 to provide compulsory universal health insurance. The program currently has over 21 million enrollees and covers all forms of health care services for 98% of the population. These databases have been used for epidemiologic research and information on prescription use, diagnoses, and hospitalizations is of high quality [9]. With approval from the NHRI, data for ambulatory care and in-patient claims, and updated registries for beneficiaries for the period 1999 to 2009 were used in this study.

Out-patient claim files include an encrypted personal and hospital identification number, date of birth, sex, date of service, and the first three International Classification of Diseases, Ninth Revision (ICD-9) codes. The hospitalization claims contain an encrypted personal and hospital identification number, date of birth, sex, length of hospital stay, date of admission and discharge, costs, the first five ICD-9 diagnostic codes, and the first five ICD-9 procedure codes. The database used in this study can be interlinked by the scrambled unique individual's personal identification number. The NHRI safeguards the privacy and confidentiality of all beneficiaries and transfers the health insurance data to health researchers after ethical approval has been obtained.

### Identification of Study Sample

This was a population-based follow-up study wherein all patients under the age of 16 years who were diagnosed with JIA, defined by the diagnosis codes of rheumatoid arthritis (ICD-9 code 714.0), rheumatoid arthritis with systemic or visceral involvement (ICD-9 code 714.2), juvenile chronic polyarthritis (ICD-9 code 714.3), ankylosing spondylitis (AS) (ICD-9 code 720.0), or psoriatic arthropathy (PsA) (ICD-9 code 696.0) between January 1, 1999 and December 31, 2009 formed the study cohort. Diagnosis of JIA by the ILAR classification criteria was widely accepted by pediatric rheumatologists in Taiwan. Enthesitis-related arthritis (ERA) was defined by the diagnostic codes of AS (ICD-9 codes 720.0) only, AS and RA (ICD-9 codes 714.0), or AS and juvenile chronic polyarthritis (ICD-9 codes 714.3). Inflammatory bowel disease (IBD)-associated arthropathy was defined by the diagnostic codes of chronic arthritis listed as above with the diagnostic codes of Crohn's disease (ICD-9 codes 555) or ulcerative colitis (ICD-9 codes 556). The IBD-associated arthropathy was included in the ERA category. Psoriatic arthropathy (PsA) was identified by ICD-9 codes 696.0. Because coding with ICD-9-CM codes of oligoarthritis (ICD-9 714.32, 714.33), polyarthritis (ICD-9 714.30 and 714.31), or systemic arthritis (ICD-9 714.2) by physicians was imprecise in the database (471 patients not coded with any subtype), patients with these three subtypes were grouped as the “JRA” group. In order to ensure diagnostic validity, JIA and IBD patients who had at least three consensus diagnoses in a year were selected.

Patients with diagnosis of systemic lupus erythematosus (ICD-9 codes 710.0) or patients who received medical treatment for less than 6 weeks were excluded. Subjects with abnormal registry claim data, such as unknown sex or birth date were also excluded. In total, there were 2636 patients with JIA in the study cohort.

### Identification of Uveitis and Complications

Uveitis was identified by ICD-9 codes 364.0 to 364.3 (iridocyclitis). Patients who had at least two consensus diagnoses in a year were selected. Cataract (ICD-9 codes 366), glaucoma (ICD-9 codes 365), blindness and low vision (ICD-9 codes 369), cystoid macular edema (ICD-9 codes 362.53), papillitis (ICD-9 codes 377.31), pan-uveitis (ICD-9 codes 360.12), posterior cyclitis (ICD-9 codes 363.21), and hypotony (ICD-9 codes 360.3) were identified. Intra-ocular surgery was identified by the ICD-9 procedure codes.

### Identification of Therapeutic Modalities

To obtain the medication record, the database was linked to the catastrophic illness database in the NHIRD. Juvenile rheumatoid arthritis was included in the list of catastrophic illnesses published by the Department of Health, Executive Yuan. The application of the status of catastrophic illness by primary care physicians was evaluated and reviewed by the Bureau of National Health Insurance. Patients with catastrophic illness certificates were eligible for exemption from insurance premiums and co-payments. Thus, data of patients with catastrophic illness patient were highly accurate and reliable.

Among the 2636 patients, 720 were linked to the catastrophic illness database using the application date for catastrophic illness certificate and acceptance number (unique for each patient). Patients with JIA who filled prescription for systemic corticosteroids, non-steroidal anti-inflammatory drugs (NSAIDs), methotrexate (MTX), azathioprine, sulfasalazine, hydroxychloroquine, cyclosporin, anti-tumor necrosis factor (TNF) agents (etanercept), topical corticosteroids, and topical mydriatics, or those who received intra-ocular surgery in the in-patient and ambulatory care order files from January 1, 1999 to December 31, 2009, were identified. The dates of prescription, daily dose, number of days supplied, number of pills per prescription, dates of intra-ocular surgery, and surgery type were noted. Patients who used systemic medications (except etanercept) for more than 28 days or ever used etanercept were defined as exposure.

### Incidence and Prevalence of JIA and JIA-associated Uveitis

The incidence rate was calculated by dividing the number of new cases by population at risk under the age of 16. Prevalence was calculated by the dividing the number of total cases by the population under the age of 16 for the corresponding year. Age- and sex-specific incidence rates were calculated by dividing the numbers of new cases in each age and sex groups by the age- and sex-specific population at risk in 2009. The population data was published by the Ministry of Internal Affairs, Executive Yuan, Taiwan.

### Statistical Analysis

Student's t test and chi square test were used for comparison between two groups. Multivariate logistic regression analysis was used to determine the risk of developing uveitis among JIA patients and risk of complications of uveitis. Odds ratios (ORs) were presented with 95% confidence interval (CI). Statistical significance was set at a two-tailed *p*<0.05. All analyses were conducted using the SAS statistical software (version 9.2, SAS Institute, Cary, NC).

## Results

### Incidence and Prevalence of JIA

**Figure 1:**
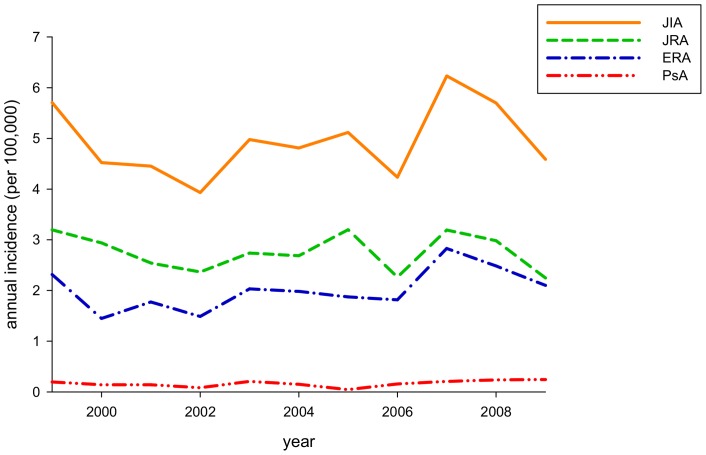
Annual incidence of juvenile idiopathic arthritis (JIA), juvenile rheumatoid arthritis (JRA), enthesitis-related arthritis (ERA), and psoriatic arthritis (PsA) for the period 1999–2009.

**Figure 2:**
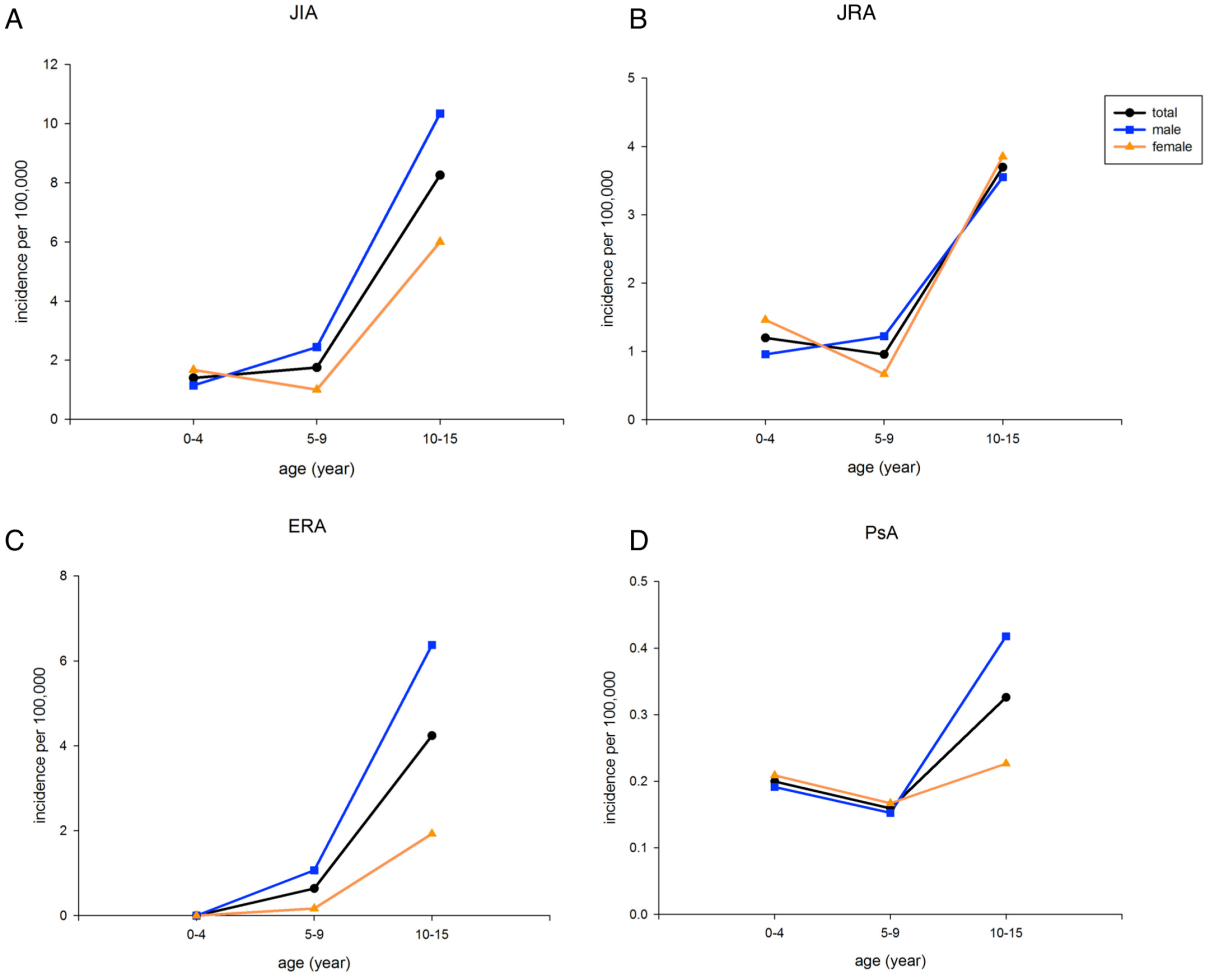
Age- and sex-specific incidence of JIA, JRA, ERA, and PsA in 2009. Incidence is higher in boys than in girls in patients with JIA aged 5–9 and 10–14 years old (male:female 2.4∶1 and 1.8∶1, respectively), ERA aged 5–9 and 10–15 years old (6.4∶1 and 3.5∶1, respectively), and PsA aged 10–15 years old (1.8∶1). Incidence is higher in females in JIA patients aged 0–4 years old (0.7∶1), JRA aged 0–4 and 10–15 years old (0.7∶1 and 0.9∶1, respectively), and PsA aged 0–9 years old (0.9∶1).

There were 2636 cases of JIA during the study period (1999–2009). The annual incidence ranged from 3.93 to 6.23 cases per 100,000 children. The average incidence of JIA, JRA, ERA, and PsA were 4.93 (male 6.0, female 3.8), 2.76, 2.01, and 0.16 per 100,000 person-years, respectively) (Figure 1, Table 1 and Table S1). Based on the age- and sex-specific incidence rate for 2009 of JIA and arthritis subtypes (Figure 2), the incidence of JIA was low for ages 0–4 and 5–9 years old, but increased with age. There was a higher female-incidence rate of JIA in age 0–4 years (male:female 0.7∶1), JRA age 0–4 years (0.7∶1), and PsA age 0–4 and 5–9 years (0.9∶1). The average prevalence of JIA, JRA, ERA, and PsA was 33.8, 20.3, 12.5, and 1.0 per 100,000 population, respectively, for the study period (Table S2).

**Table 1:** Demographic data of patients with JIA.

	JIA n = 2636	JRA n = 1520	ERA n = 1033	PsA n = 83
%	100%	57.7%	39.2%	3.2%
Male-female ratio	1.69∶1	1.17∶1	3.3∶1	1.02∶1
Diagnosis age (yr) (min-max)	11.5±3.7 (0.6–15.99)	10.2±3.9 (0.6–15.97)	13.4±2.3 (3.6–15.99)	11.9±3.8 (1.4–15.96)
Male	11.7±3.6^a^	10.0±3.8	13.5±2.3	11.5±4.1
Female	11.2±3.9^a^	10.4±4.0	13.3±2.5	12.4±3.4
Follow-up duration (yr) (min-max)	3.5±3.5 (0.02–14.8)	3.5±3.7 (0.03–14.8)	3.6±3.3 (0.02–14.6)	2.6±3.0 0.02–10.6)
Male	3.8±3.6^b^	3.7±3.8^c^	3.8±3.4^d^	2.7±3.3
Female	3.1±3.4^b^	3.2±3.5^c^	2.7±3.0^d^	2.4±2.6
uveitis	125/2636 (4.7%)	44/1520 (2.9%)	81/1033 (7.8%)	0
Male	87/1656 (5.3%)	21/822 (2.6%)	66/793 (8.3%)	0
Female	38/980 (3.9%)	23/699 (3.3%)	15/240 (6.3%)	0
cataract	14/125 (11.1%)	9/44 (20.5%)	5/81 (6.2%)	0
Male	4/87 (4.6%)	1/21 (4.8%)	3/66 (4.6%)	0
Female	10/38 (26.3%)	8/23 (34.8%)	2/15 (13.3%)	0
glaucoma	31/125 (24.8%)	10/44 (22.7%)	21/81 (25.9%)	0
Male	21/87 (24.1%)	2/21 (9.5%)	19/66 (28.8%)	0
Female	10/38 (26.3%)	8/23 (34.8%)	2/15 (13.3%)	0
Blindness and low vision	0	0	0	0

a *p* = 0.001, ^b^ *p*<0.0001, ^c^ *p* = 0.01, and ^d^ *p*<0.0001 compared male and female patients, by student's t test.

Abbreviations: JIA, juvenile idiopathic arthritis; JRA, juvenile rheumatoid arthritis; ERA, enthesitis-related arthritis; PsA, and psoriatic arthritis.

### Clinical Features of Patients with JIA and JIA-associated Uveitis

Patients with JIA were divided into three groups: 1520 (57.7%) JRA, 1033 (39.2%) ERA, and 83 (3.1%) PsA (Table 1). Male-to-female ratio was 1.69∶1 in JIA and 3.3∶1 in ERA. The mean age at diagnosis was older in patients with ERA (13.4±2.3 years). The mean follow-up duration of JIA was 3.5±3.5 years, with significantly longer duration in male patients (*p*<0.0001). Of the five patients with IBD-associated arthropathy in the study cohort, one had ulcerative colitis and four had Crohn's disease.

**Figure 3:**
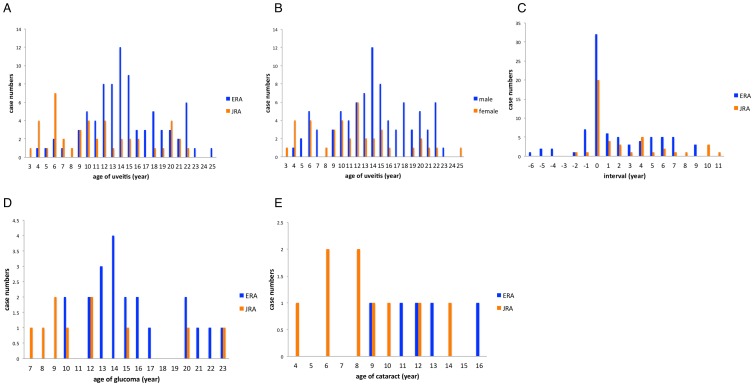
(A and B) Distribution of diagnostic age of uveitis in 125 uveitis patients considering JIA subtype and sex. The mean age of uveitis was 15.1±4.4 years for patients with ERA, 11.8±5.6 years for JRA, 14.7±4.8 years for males, and 12.1±5.5 years for females. (**C**) Distribution of time interval for 125 patients considering JIA subtype. In the means time elapsed between diagnosis of JIA and uveitis, with 0 being the point at which JIA was diagnosed, patients with negative elapsed time were diagnosed as having uveitis before they were diagnosed as having JIA. (**D**) Distribution of age of glaucoma in uveitis patients considering JIA subtype. The median age of glaucoma was 11.6 years for patients with JRA and 14.7 years for those with ERA. (**E**) Distribution of age of cataract in uveitis patients considering JIA subtype. The median age of cataract was 8.4 years for patients with JRA and 12.2 years for those with ERA.

Uveitis (all anterior uveitis) occurred in 125 (4.7%) patients with JIA, in 44 (2.9%) with JRA, and in 81 (7.8%) with ERA, with male predominance (87/125, 69.9%) (Table 1). None of the patients with IBD-associated arthropathy had uveitis. Three patients with pan-uveitis had posterior segment manifestations. The annual incidence of JIA-associated uveitis increased from 0.16 cases per 100,000 children in 1999 to 0.37 cases per 100,000 children in 2009 (average 0.25 cases per 100,000 children) (Table S1). The mean age at diagnosis of uveitis and JIA were 13.4±5.1 years (range, 3.0–25.0 years) and 11.9±3.7 years (range, 2.0–15.98 years), respectively, in 125 patients. The age at diagnosis of uveitis was significantly older in patients with ERA (15.1±4.4 years) than in patients with JRA (11.8±5.6 years), (*p* = 0.0004) (Figure 3A). Uveitis developed within the same year of JIA diagnosis in 52 patients (41.6%) but developed more than one year before JIA diagnosis in 15 patients (12%), especially those with ERA (Figure 3C). In 49 (39.2%) patients (10 JRA patients and 39 ERA patients) with uveitis diagnosed before JIA, the mean interval between diagnosis of JIA and uveitis was −1.0±1.6 years. The mean interval between diagnosis of JIA and uveitis was 3.9±3.2 years in 76 (60.8%) patients with uveitis diagnosed after JIA (Table S3).

In the 125 patients with JIA-associated uveitis, complications included cataract (14, 11.2%), glaucoma (31, 24.8%), and cystoid macular edema with disc edema (1, 0.8%). More female patients with uveitis (10/38, 26.3%) had complications with cataract than male patients (4/87, 4.6%) (Table 1). Patients with uveitis diagnosed before JIA had higher rates of glaucoma (18/49, 36.7%) and cataract (10/49, 20.4%). The mean follow-up duration for uveitis was 1.9±2.7 years (range, 0.1–10.4 years). None of the patients with uveitis developed blindness or low vision. Five (4%) patients with uveitis who received intra-ocular surgeries include one who underwent trabeculectomy, three who underwent cataract extraction and intra-ocular lens implantation, and one who underwent combined lens extraction, vitrectomy, and cyclophoto-coagulation.

The therapeutic modalities were analyzed from 720 JIA patients by linking to the catastrophic illness database. Systemic corticosteroids (80.7%), NSAIDs (99.6%), MTX (62.2%), and sulfasalazine (52.4%) were commonly used in JIA patients. Etanercept was used in 104 of 720 (14.4%) patients with JIA and in 7 of 37 (18.9%) patients with uveitis. Among patients with uveitis, 54.1% used topical corticosteroids longer than 28 days (Table S4).

### Risk Factors and Complications of Uveitis

Multivariate logistic regression analyses were performed to investigate the risk factors of uveitis and its complications among patients with JIA. The ERA subtype was significantly associated with uveitis (OR: 3.47; 95% CI: 2.24–5.37) (*p*<0.0001), after adjustments for sex and age of diagnosis of JIA (Table S5). Uveitis diagnosed before JIA was the most significant risk factor for complications of uveitis (OR: 3.54; 95% CI: 1.44–8.72) in patients with JIA-associated uveitis. In patients with JRA-associated uveitis, both female (OR: 7.08; 95% CI: 1.08–46.51) and uveitis diagnosed before JIA (OR: 7.82; 95% CI: 1.35–45.18) were significant risk factors for complications (Table 2).

**Table 2:** Risk factors for complications of glaucoma or cataract in uveitis patients.

	JIA-associated uveitis (n = 125)			JRA-associated uveitis (n = 44)		
	OR	95% CI	*p* value	OR	95% CI	*p* value
Female	1.42	0.56–3.57	0.45	7.82	1.35–45.18	0.02
JRA	1.54	0.56–4.24	0.40	-	-	-
Arthritis diagnosed <6 years	0.92	0.20–4.22	0.92	1.32	0.21–8.25	0.77
Uveitis diagnosed before JIA	3.54	1.44–8.72	0.006	7.08	1.08–46.51	0.04

Abbreviations: JIA, juvenile idiopathic arthritis; JRA, juvenile rheumatoid arthritis.

## Discussion

This study is the first nationwide population-based study describing the incidence and prevalence of JIA and uveitis in Asia. Notably, this research is one of the few population-based studies that collected diagnostic information from birth onwards, with a follow-up period of more than ten years. The coverage rate of NHIP for children is more than 99% and the vast majority (over 99%) general hospitals have contracts with NHIP during the study period. Given the low incidence of JIA in childhood, this large nationwide cohort makes is possible to estimate the incidence of JIA-associated uveitis. In this study, the average incidence of JIA is 4.93 cases per 100,000 population per year and the prevalence is 33.8 per 100,000 population. Smaller studies from Asia show a low disease prevalence of JIA [10], [11]. The prevalence of JCA in Taiwan is 3.8 per 100,000 (95% CI: 3.3–4.3) previously [11]. Since epidemiologic studies vary enormously as regards study methodologies (including different classification criteria) and populations, direct comparison of the incidence between studies is limited [12]. Comparing epidemiologic studies of JIA, the annual incidence is 2.6 to 23 per 100,000 children per year and the prevalence is estimated to be 15.7–140 per 100,000 children [13]–[19]. The highest JIA incidence rates are found in some Scandinavian countries. The incidence rate in Taiwan is close to 6.9 cases per 100,000 population in Spain [13] and is higher than the incidence rate of 0.83 per 100,000 for JRA in Japan in 1997 [10].

Epidemiologic studies of JIA from countries outside Europe and North America show different patterns of prevalence for JIA subtypes and different rates of development of uveitis, which may be due to ethnic differences [20], [21]. In North America and Europe, the most common JIA subtype is oligoarticular (30–60%), with peak age of onset at 2–4 years. Female predominance (60–70%), younger onset age of arthritis, and lower frequency of ERA (7–13%) are more frequently reported in studies in Western countries [14], [20]–[25]. Patients of African American or native American descent are more likely to have RF-positive polyarticular JIA, with peak age of onset at 6–12 years [21]. In contrast, ERA is most common in Asia based on studies in Taiwan, Canada, and India [20], [26], [27]. Shen et al. reported 195 children with JIA diagnosed between 1995 and 2010 in a tertiary center in Taiwan, with 37.4% ERA and 32.3% HLA-B27-positivity [26]. A high frequency of ERA (24%) and less oligoarticular JIA were also observed in JIA children of Asian origin in a large multi-ethnic cohort study in Canada [20]. The present study shows a low incidence of JIA 1.4 (male 1.1, female 1.7) per 100,000 children age at 1–4 years, which is compatible with the low frequency of oligoarticular JIA (23.1%) and an older age of disease onset (mean age, 6.3 years) in Shen's study [26]. Moreover, the presence of associations between the human leukocyte antigen (HLA) and non-HLA gene polymorphisms and susceptibility to different JIA subtypes has been found mostly in Caucasians [21], [28]. Further genome-wide association studies of patients with JIA in Han Chinese are warranted to provide further insights into the genetic basis of ethnic difference in the development of JIA.

The prevalence of ERA in the present study is 12.5 per 100,000 children, which is far less than the prevalence of spondyloarthropathies among Taiwanese Chinese adults, which is about 0.2–0.4%. The prevalence of HLA-B27 varies markedly around the world, 24% in northern Scandinavians, 8% in Caucasians, 4% in North Africans, 2–9% in Chinese, and 0.1–0.5% in Japanese [29], [30]. The high proportion of ERA in this cohort explains the older diagnostic age of JIA (11.5±3.7 years), male predominance (62.8%), and the longer follow-up duration in males (3.8±3.6 years) compared to female patients (3.1±3.4 years). Studies have shown that ERA with HLA-B27 positivity tends to have a continuously active or relapsing course, and a poorer outcome compared to oligoarticular or polyarticular JIA, which requires a longer follow-up duration [26], [31]–[33].

The estimated prevalence of uveitis in patients with JIA ranges from 4–38% among different studies worldwide. The cumulative incidence of uveitis in JRA varies according to geographic location, highest in Scandinavia, then the US, followed by Asia, and lowest in India [34], [35]. Compared to a recent report showing the incidence of uveitis in children of 39.7 cases per 100,000 person-year in Taiwan using the NHIRD since 2003 [36], JIA-associated uveitis is rare (0.25 cases per 100,000 person-year) in Taiwanese children. Uveitis develops in 4.7% and 7.8% of patients with JIA and ERA, respectively. The results here are close to the study by Shen et al. that uveitis occurs in 6.7% and 9.6% of patients with JIA and ERA, respectively [26].

In the 125 patients with JIA-associated uveitis, 89.6% are boys and 64.8% are ERA-associated uveitis. In contrast to the asymptomatic chronic iridocyclitis seen with other JIA subtypes, particularly ANA-positive girls with early onset oligoarthritis, uveitis in ERA patients is often symptomatic (redness and pain), has sudden onset, and is more common in older adolescents and adults [37]. Male predominance is also found in ERA or spondyloarthritis with HLA-B27 positivity [5], [30]. However, the increased risk for uveitis (OR 3.47) in ERA in this study is not seen in previous studies, partly due to a lower percentage of ERA in their cohort.

Although 39.2% of uveitis developed before the diagnosis of JIA, 15 (12%) patients developed uveitis more than one year before JIA diagnosis, while about 41.6% of uveitis developed within the same year of JIA diagnosis. In previous reports, 75% of patients develop uveitis within 1 year and 90% by 4 years after arthritis onset [25]. Uveitis manifesting before arthritis is rare (2.5–10%) in JIA, but uveitis is frequently the first sign of previous undiagnosed spondyloarthropathies in 30–40% patients with acute anterior uveitis [25], [38], [39]. Acute anterior uveitis may precede joint symptoms by months or years in HLA-B27 positive boys with ERA. On the other hand, if asymptomatic uveitis is diagnosed before JIA, it is often severe [5]. The median interval from onset of symptoms of arthritis to first pediatric rheumatology assessment is 2.6 to 10 months (range, 0–163.2 months) in different countries around the world [40]. Such information is lacking in Taiwan. Understanding the interval from onset of uveitis or arthritis to diagnosis, as well as the underlying complex reasons during the pathway of referral to pediatric rheumatologists, is important. Collaboration between ophthalmologists and rheumatologists greatly aids the prompt diagnosis and treatment of such patients.

High complication rates have been previously reported for JIA uveitis, higher than uveitis of other etiologies [8], [25]. Although the inflammation of JIA-associated uveitis is primarily anterior in nature, chronic active inflammation can result in permanent structural damage in the posterior part of the eye. Complications of JIA-associated uveitis include secondary glaucoma (33%), cataract (78%), synechia (58%), retinal edema (29%), and visual impairment [5], [6], [8], [41]. Cystoid macular edema (multi-cystic collection of fluid within the macula) is an important cause of visual impairment. Visual loss to 20/200 or less is seen in 14–18% of children with JIA, but not in the cases here [5], [25], [42].

The mainstay of treatment for uveitis is local therapy with corticosteroids. Systemic treatment with NSAIDs, disease-modifying anti-rheumatic drugs (including MTX, cyclosporine, azathioprine), systemic glucocorticoids, and anti-TNF agents are also useful. Use of immuno-suppressive drugs has been associated with reduced risk of vision loss in approximately 60% of JIA-associated uveitis [43].

The long-term prognosis of JIA-associated uveitis is continuously improving due to routine screening and advances in the medical and surgical treatment of both arthritis and uveitis. The American Academy of Pediatrics recommends screening for uveitis in children with JRA based on the type of arthritis, age of onset of arthritis, disease duration, and ANA positivity. High-risk groups with oligoarthritis or polyarthritis, age <7 years old at onset of arthritis, duration <4 years, and positive ANA need slit-lamp examination every 3 months until adolescence [44]. A visit with an ophthalmologist may be worth considering for patients with ERA, even if only on a yearly basis or if symptomatic [25]. The present study shows that uveitis before arthritis has a higher risk of complications, which frequently occur in such patients due to delays in the diagnosis of the insidious ocular condition. In contrast, male sex, shorter duration of uveitis, older age at disease onset, and shorter delay in presentation to an experienced ophthalmologist are associated with improved visual acuity [5].

This study has several limitations. First, the validity of diagnosis of JIA and subtypes cannot be re-confirmed. There is no direct evidence regarding clinical validity of diagnosis for JIA and JIA subtypes. However, validity should be satisfactory given that the vast majority of JIA cases have been first diagnosed in hospitals (including medical centers, regional hospital, and district hospital). The diagnostic accuracy is enhanced by the study design of selecting patients with at least three consensus diagnoses in a year and with continuous medication use for 6 weeks. The results are also compatible to other studies in Taiwan using the current selection and exclusion criteria of JIA. Undifferentiated arthritis cannot be analyzed in a separate category based on the diagnostic codes. According to Shen's study, undifferentiated arthritis is present in 5/195 (2.6%) JIA cases. Second, therapeutic modalities are only analyzed in 720 patients with JIA. There is no adequate sample size to analyze treatment outcomes according to the specific immuno-suppressive agent used. Third, manifestations of uveitis (symptomatic or asymptomatic at presentation) and visual acuity are not included in the database. Additional large-scale cohort studies are warranted to describe patients with JIA regarding subtypes of classification, manifestations (including the heterogeneous presentation of ERA and visual acuity in uveitis), treatment, and complications of uveitis.

In conclusion, the incidence rate of JIA has not changed significantly in the past ten years. The incidence of JIA-associated uveitis has increased gradually. Higher percentage of males in patients with ERA and the strong association between ERA and uveitis are unique for children with JIA in Taiwan. Uveitis diagnosed before arthritis is an important risk factor for complications. Long-term follow-up for those with uveitis of unknown cause is needed for the possibility of developing JIA several years later. The screening protocols of uveitis are not standardized among hospitals in Taiwan. This study may facilitate the optimization of guidelines for the screening and follow-up of JIA patients with uveitis in Taiwan.

## Supporting Information

Table S1 **Annual incident cases and incidence of JIA and JIA-associated uveitis by calendar year.**
(DOCX)

Table S2 **Prevalence of JIA and JIA subtypes by calendar year (cases per 100,000 population).**
(DOCX)

Table S3 **Clinical features of 125 patients with JIA-associated uveitis.**
(DOCX)

Table S4 **Therapeutic modalities of 720 patients with JIA linked to catastrophic illness database in 1999 to 2009.**
(DOCX)

Table S5 **Risk factors for uveitis in patients with JIA.**
(DOCX)
