# Supplementary material for: Juvenile Idiopathic Arthritis-Associated Uveitis: A Nationwide Population-Based Study in Taiwan
Source: PLoS One. 2013 Aug 5;8(8):e70625. doi: 10.1371/journal.pone.0070625 (PMC3734244; doi:10.1371/journal.pone.0070625)
Supplement: Table S1 — Annual incident cases and incidence of JIA and JIA-associated uveitis by calendar year. (DOCX) [file pone.0070625.s001.docx]

**Table S1.** Annual incident cases and incidence of JIA and JIA-associated uveitis by calendar year

| Year | Population at risk (<16 years) | Incident cases of JIA | Annual incidence of JIA | Incident cases of uveitis | Annual incidence of JIA-associated uveitis |
| --- | --- | --- | --- | --- | --- |
| 1999 | 5098517 | 291 | 5.71 | 8 | 0.16 |
| 2000 | 5040737 | 228 | 4.52 | 9 | 0.18 |
| 2001 | 4961819 | 221 | 4.45 | 10 | 0.20 |
| 2002 | 4906891 | 193 | 3.93 | 9 | 0.18 |
| 2003 | 4821219 | 240 | 4.98 | 6 | 0.12 |
| 2004 | 4695748 | 226 | 4.81 | 14 | 0.30 |
| 2005 | 4591928 | 235 | 5.12 | 13 | 0.28 |
| 2006 | 4461958 | 189 | 4.24 | 15 | 0.34 |
| 2007 | 4348525 | 271 | 6.23 | 16 | 0.37 |
| 2008 | 4228940 | 241 | 5.70 | 10 | 0.24 |
| 2009 | 4097559 | 188 | 4.59 | 15 | 0.37 |

Annual incidence represents the number of incident cases divided into population at risk (<16 years) at the beginning of that year (1/100,000 population)
