# Supplementary material for: Juvenile Idiopathic Arthritis-Associated Uveitis: A Nationwide Population-Based Study in Taiwan
Source: PLoS One. 2013 Aug 5;8(8):e70625. doi: 10.1371/journal.pone.0070625 (PMC3734244; doi:10.1371/journal.pone.0070625)
Supplement: Table S2 — Prevalence of JIA and JIA subtypes by calendar year (cases per 100,000 population). (DOCX) [file pone.0070625.s002.docx]

**Table S2.** Prevalence of JIA and JIA subtypes by calendar year (cases per 100,000 population)

| year | JIA | | |  | JRA | | |  | ERA | | |  | PsA | | |
| --- | --- | --- | --- | --- | --- | --- | --- | --- | --- | --- | --- | --- | --- | --- | --- |
|  | T | M | F |  | T | M | F |  | T | M | F |  | T | M | F |
| 1999 | 7.9 | 10.0 | 5.6 |  | 5.2 | 6.0 | 4.4 |  | 2.5 | 3.7 | 1.1 |  | 0.2 | 0.3 | 0.1 |
| 2000 | 12.5 | 15.5 | 9.3 |  | 8.2 | 9.0 | 7.4 |  | 4.0 | 6.0 | 1.7 |  | 0.3 | 0.4 | 0.2 |
| 2001 | 17.2 | 20.6 | 13.5 |  | 10.9 | 11.6 | 10.1 |  | 5.8 | 8.4 | 3.0 |  | 0.5 | 0.6 | 0.3 |
| 2002 | 21.3 | 25.7 | 16.6 |  | 13.4 | 14.4 | 12.3 |  | 7.4 | 10.6 | 3.9 |  | 0.6 | 0.7 | 0.4 |
| 2003 | 26.7 | 32.3 | 20.5 |  | 16.4 | 17.5 | 15.2 |  | 9.5 | 14.0 | 4.7 |  | 0.8 | 0.9 | 0.7 |
| 2004 | 32.2 | 38.6 | 25.2 |  | 19.5 | 20.4 | 18.5 |  | 11.8 | 17.3 | 5.7 |  | 1.0 | 1.0 | 0.9 |
| 2005 | 38.0 | 45.3 | 30.1 |  | 23.1 | 23.9 | 22.3 |  | 13.9 | 20.4 | 6.8 |  | 1.0 | 1.0 | 1.0 |
| 2006 | 43.4 | 52.2 | 33.8 |  | 26.1 | 27.3 | 24.7 |  | 16.1 | 23.6 | 7.9 |  | 1.2 | 1.2 | 1.2 |
| 2007 | 50.7 | 60.5 | 40.1 |  | 29.9 | 30.9 | 28.9 |  | 19.4 | 28.3 | 9.6 |  | 1.4 | 1.3 | 1.6 |
| 2008 | 57.9 | 69.6 | 45.1 |  | 33.7 | 35.1 | 32.3 |  | 22.4 | 32.9 | 11.0 |  | 1.7 | 1.6 | 1.8 |
| 2009 | 64.3 | 77.5 | 49.9 |  | 37.1 | 38.4 | 35.6 |  | 25.2 | 37.1 | 12.2 |  | 2.0 | 2.0 | 2.1 |
| Average | 33.8 | 40.7 | 26.3 |  | 20.3 | 21.3 | 19.2 |  | 12.5 | 18.4 | 6.2 |  | 1.0 | 1.0 | 0.9 |

T=total, M=male, F=female
