# Supplementary material for: Juvenile Idiopathic Arthritis-Associated Uveitis: A Nationwide Population-Based Study in Taiwan
Source: PLoS One. 2013 Aug 5;8(8):e70625. doi: 10.1371/journal.pone.0070625 (PMC3734244; doi:10.1371/journal.pone.0070625)
Supplement: Table S3 — Clinical features of 125 patients with JIA-associated uveitis. (DOCX) [file pone.0070625.s003.docx]

**Table S3.** Clinical features of 125 patients with JIA-associated uveitis

|  | Uveitis (n=125) |
| --- | --- |
| Male | 87 (69.6%) |
| JRA | 44 (35.2%) |
| ERA | 81 (64.8%) |
| Diagnostic age of JIA (yr) | 11.9±3.7 (2.02-15.98) |
| Diagnostic age of uveitis (yr) | 13.4±5.1 (3.0-25.0) |
| Interval between diagnosis of JIA and uveitis (yr) |  |
| Uveitis before JIA | -1.01±1.57 |
| Uveitis after JIA | 3.93±3.15 |
| Uveitis before diagnosis of JIA | 49/125 (39.2%) |
| JRA | 10/44 (22.7%) |
| ERA | 39/81 (49.2%) |
| Complicated with glaucoma | 18/49 (36.7%) |
| Complicated with cataract | 10/49 (20.4%) |
| Uveitis follow-up duration (yr) | 1.9±2.7 (0.1-10.4) |
| JRA | 1.4±2.2 |
| ERA | 2.2±2.9 |

Data are expressed as mean ± standard deviation (range)
