# Supplementary material for: Juvenile Idiopathic Arthritis-Associated Uveitis: A Nationwide Population-Based Study in Taiwan
Source: PLoS One. 2013 Aug 5;8(8):e70625. doi: 10.1371/journal.pone.0070625 (PMC3734244; doi:10.1371/journal.pone.0070625)
Supplement: Table S4 — Therapeutic modalities of 720 patients with JIA linked to catastrophic illness database in 1999 to 2009. (DOCX) [file pone.0070625.s004.docx]

**Table S4.** Therapeutic modalities of 720 patients with JIA linked to catastrophic illness database in 1999 to 2009.

|  |  | JIA |  |  | JIA | |  | JIA uveitis | |
| --- | --- | --- | --- | --- | --- | --- | --- | --- | --- |
|  | All (n=720) | JRA (n=631) | ERA (n=89) |  | Without uveitis (n=683) | With uveitis  (n=37) |  | With complication (n=3) | Without complication (n=34) |
| Corticosteroids | 581 (80.7) | 505 (80.0) | 76 (85.4) |  | 549 (80.4) | 32 (86.5) |  | 3 (100) | 29 (85.3) |
| NSAIDs | 717 (99.6) | 628 (99.5) | 89 (100) |  | 681 (99.7) | 36 (97.3) |  | 2 (66.7) | 34 (100) |
| Methotrexate | 448 (62.2) | 387 (61.3) | 61 (68.5) |  | 421 (61.6) | 27 (73.0) |  | 2 (66.7) | 25 (73.5) |
| Azathioprine | 155 (21.5) | 137 (21.7) | 18 (20.2) |  | 148 (21.7) | 7 (18.9) |  | 1 (33.3) | 6 (17.7) |
| Salfasalazine | 377 (52.4) | 307 (48.7) | 70 (78.7) |  | 355 (52.0) | 22 (59.5) |  | 1 (33.3) | 21 (61.8) |
| Hydroxychloroquine | 213 (29.6) | 188 (29.8) | 25 (28.1) |  | 206 (30.2) | 7 (18.9) |  | 0 (0) | 7 (20.6) |
| Cyclosporin | 105 (14.6) | 92 (14.6) | 13 (14.6) |  | 101 (14.8) | 4 (10.8) |  | 0 (0) | 4 (11.8) |
| Etanercept | 104 (14.4) | 91 (14.4) | 13 (14.6) |  | 97 (14.2) | 7 (18.9) |  | 1 (33.3) | 6 (17.7) |
| Topical steroids |  |  |  |  |  |  |  |  |  |
| Ever use | 554 (74.6) | 482 (76.4) | 72 (80.9) |  | 522 (76.4) | 32 (86.5) |  | 3 (100) | 29 (85.3) |
| Use > 28 days | 108 (15) | 95 (15.1) | 13 (14.6) |  | 88 (12.9) | 20 (54.1) |  | 3 (100) | 17 (50) |
| Topical mydriatics |  |  |  |  |  |  |  |  |  |
| Ever use | 471 (65.4) | 411 (65.1) | 60 (67.4) |  | 441 (64.6) | 30 (81.1) |  | 3 (100) | 27 (79.4) |
| Use > 28 days | 138 (19.2) | 122 (19.3) | 16 (18.0) |  | 122 (17.9) | 16 (43.2) |  | 3 (100) | 13 (38.2) |

Number (%)

Systemic medication (except etanercept) used > 28 days as user; etanercept ever used as user
