# Supplementary material for: Juvenile Idiopathic Arthritis-Associated Uveitis: A Nationwide Population-Based Study in Taiwan
Source: PLoS One. 2013 Aug 5;8(8):e70625. doi: 10.1371/journal.pone.0070625 (PMC3734244; doi:10.1371/journal.pone.0070625)
Supplement: Table S5 — Risk factors for uveitis in patients with JIA. (DOCX) [file pone.0070625.s005.docx]

**Table S5.** Risk factors for uveitis in patients with JIA

|  | OR | 95% CI | P value |
| --- | --- | --- | --- |
| Male | 1.04 | 0.69-1.55 | 0.87 |
| ERA | 3.47 | 2.24-5.37 | <0.0001 |
| 0<diagnosis age<5 | 1.0 | - | - |
| 5 ≤ diagnosis age<10 | 0.89 | 0.39-2.00 | 0.77 |
| 10≤diagnosis age<16 | 0.55 | 0.25-1.22 | 0.14 |
